# Organic-sulfur-zinc hybrid nanoparticle for optical applications synthesized via polycondensation of trithiol and Zn(OAc)_2_

**DOI:** 10.1186/1556-276X-8-373

**Published:** 2013-09-02

**Authors:** Bungo Ochiai, Hirohisa Konta

**Affiliations:** 1 Department of Chemistry and Chemical Engineering, Faculty of Engineering, Yamagata University, 4-3-16 Jonan, Yamagata, Yonezawa 992-8510, Japan

**Keywords:** Organic–inorganic hybrid, Nanoparticle, Refractive index, Sulfur, Zinc

## Abstract

Organic-sulfur-zinc hybrid materials were prepared via polycondensation of Zn(OAc)_2_ and trithiols bearing various alkyl groups. A soluble nanoparticle could be obtained by the polycondensation using a trithiol bearing octadecyl moieties. The good dispersing ability as nano-scaled particles was confirmed by dynamic light scattering and atomic force microscopy analyses. This hybrid nanoparticle was miscible with poly(methyl methacrylate) and served as a refractive additive to increase refractive indexes. The calculated refractive index value for the nanoparticle was 1.58.

## Background

Excellent high refractive index materials are demanded by recent rapid development of mobile devices, solar cells, and luminescent devices. Various materials have been developed by hybridization of organic and inorganic materials, complementing the properties of each component. For example, organic materials provide flexibility and easy processing, and inorganic materials provide optical and mechanical properties. Typical preparation methods for organic–inorganic hybrids are incorporation of metal oxide into polymer matrices via sol–gel methods [1-3] and mixing of polymers and nanoparticles of metal oxides [3-8] or sulfides [9,10]. However, both of the methods contain some disadvantages. Sol–gel methods realized facile and green procedures but are typically time consuming and accompanied by shrinkage during drying processes. Mixing of nano-scaled metal compounds is advantageous by the fast process, but specific coating and precise tuning of the reaction conditions are required for the preparation of nano-scaled metal compounds.

Another approach to conquer these problems is the use of organometallic materials [11]. Ene-thiol polyaddition of dithiols with tetravinyl-silane, germane, and tin gave polymers with high refractive indexes ranging from 1.590 to 1.703 and excellent physical properties.

Encouraged by this work, we designed new organic–inorganic hybrid materials based on sulfur as a bridge for organic and inorganic components, namely organic-sulfur-inorganic hybrid materials. The important character of sulfur for this approach is the ability to form stable linkages with both organic and inorganic structures. Another beneficial character of sulfur is its high atom refraction, by which sulfur has served as an important component for optical materials [12-17]. This bridging ability has been mostly applied for the functionalization of inorganic surfaces with organic structures such as the modification of gold surface [18-20] and quantum dots [21,22] with thiols. Although many stable metal thiolates have been reported [23-27], these compounds have not been applied as optical materials as far as we know. As the metal for this approach, zinc was selected because of its high refractivity and low toxicity. We employed trithiols (TSHs) obtained from a trifunctional dithiocarbonate and amines, whose structures can be easily tuned by the substituent on the amines [28,29]. Polycondensation of TSHs with Zn(OAc)_2_ yielded organic-sulfur-inorganic hybrid nanoparticles serving as refractive ingredients for poly(methyl methacrylate) (PMMA).

## Methods

### Materials

1,4-Dioxane was dried over sodium and distilled under a nitrogen atmosphere prior to use. A trifunctional cyclic dithiocarbonate, 1,3,5-tris(2-thioxo-1,3-oxathiolan-5-yl)methyl)-1,3,5-triazinane-2,4,6-trione (TDT), was prepared as reported [23]. Other reagents were used as received.

### Measurements

^1^H and ^13^C nuclear magnetic resonance (NMR) spectra were measured on a JEOL ECX-400 instrument (Tokyo, Japan) using tetramethylsilane as an internal standard (400 MHz for ^1^H and 100 MHz for ^13^C). Fourier transform infrared spectra were measured on a Horiba FT-210 instrument (Kyoto, Japan). Size exclusion chromatography measurements were performed on a Tosoh HLC-8220 GPC (Tokyo, Japan) equipped with Tosoh TSK-gel superAW5000, superAW4000, and superAW3000 tandem columns using tetrahydrofuran (THF) with a flow rate of 1.0 mL/min as an eluent at 40°C. Quantitative elemental analysis was performed with a system consisting of a JEOL JSM6510A scanning electron microscope equipped with a JEOL JED2300 energy dispersive X-ray (EDX) spectrometer operated at an acceleration voltage of 20 kV. The samples were compressed as flat tablets, and the atom ratios were calculated as averages of data obtained from ten spots. Refractive indexes (*n*_D_s) were measured with an Atago DR-A1 digital Abbe refractometer (Tokyo, Japan). Dynamic light scattering (DLS) measurements were performed using a Malvern Zetasizer nano-ZS instrument (Worcestershire, UK) equipped with a 4-mW He-Ne laser (633 nm) and 12-mm square glass cuvettes at 25°C. The samples were dissolved in anhydrous THF (1.3 g/L). Atomic force microscopic (AFM) measurements were performed on an Agilent 5500 atomic force microscope (Santa Clara, CA, USA) operated in tapping mode. The samples were spin cast on freshly cleaved mica substrates from anhydrous THF solutions.

### Experimental methods

#### Synthesis of TSHs (typical procedure)

TSHs were prepared according to the previous report [29]. The synthetic procedure for a trithiol bearing octadecyl chains (OTSH) is as follows. Octadecylamine (1.62 g, 6.02 mmol), TDT (1.05 g, 2.00 mmol), and THF (5.0 mL) were added to a round-bottom flask, and the mixture was stirred at room temperature for 24 h. Volatile substances were evaporated off, and the residue was purified using SiO_2_ gel column chromatography, eluted with EtOAc/hexane (*v*/*v* = 1/10). OTSH was obtained as a white solid (2.03 g, 1.52 mmol, 76.0%).

^1^H-NMR (CDCl_3_/CF_3_CO_2_H = 5:1, rt, % *δ* in ppm): 0.88 (9H, t, *J* = 7.0 Hz, -C*H*_*3*_), 1.27 to 1.31 (93H, -(C*H*_*2*_)_15_CH_3_ and -S*H*), 1.56 to 1.65 (6H, m, -CH_2_C*H*_*2*_(CH_2_)_15_-), 2.92 (6H, m, -CHC*H*_*2*_SH), 3.30 to 3.41 (6H, m, -NHC*H*_*2*_CH_2_-), 4.11 to 4.46 (6H, m, -NC*H*_*2*_CH-), 5.75 (3H, br, -CH_2_C*H*O-), 8.06 (3H, br, -(C=S)N*H*CH_2_-). ^13^C-NMR (CDCl_3_/CF_3_CO_2_H = 5:1, *δ* in ppm): 13.76 (−CH_2_*C*H_3_), 22.64 (−(CH_2_)_15_*C*H_2_CH_3_), 25.90 to 27.26 (−*C*H_2_SH), 28.76 to 31.93 (−CH_2_(*C*H_2_)_15_CH_2_-), 44.35 (−NH*C*H_2_(CH_2_)_15_-), 45.91 (−N*C*H_2_CH-), 77.44 (−CH_2_*C*HO-), 149.29 (*C*=O), 188.55 (*C*=S). IR (KBr, cm^−1^): 3,320 (NH), 2,575 (SH), 1,691 (C=O), 1,165 (C=S), 1,049 (C=S).

*BTSH*. TSH with benzyl moieties was prepared using benzylamine (643 mg, 6.01 mmol) and TDT (1.05 g, 1.99 mmol) in a similar manner with OTSH (1.43 g, 1.68 mmol, 84.4%).

^1^H-NMR (CDCl_3_/CF_3_CO_2_H = 5:1, rt, *σ* in ppm): 1.32 (3H, br, -S*H*), 2.82 (6H, br, -C*H*_*2*_SH), 4.06 to 4.47 (6H, br, -NHC*H*_*2*_Ar), 4.47 to 4.57 (6H, br, -C*H*_*2*_CH(CH_2_SH)O-), 5.73 (3H, br, -CH_2_C*H*(CH_2_SH)O-), 7.25 to 7.36 (15H, m, *Ar*), 8.35 (3H, br, -N*H*-). ^13^C-NMR (CDCl_3_/CF_3_CO_2_H = 5:1, rt, *σ* in ppm): 25.98 (−*C*H_2_SH), 45.37 (−*C*H_2_CH(CH_2_SH)O-), 47.58 (−NH*C*H_2_Ar), 79.52 (−CH_2_*C*H(CH_2_SH)O-), 127.49 to 135.80 (−CH_2_*Ar*), 149.48 (*C*=O), 187.99 (*C*=S). IR (KBr, cm^−1^): 3,348 (NH), 2,573 (SH), 1,695 (C=O), 1,165 (C=S).

*HTSH*. TSH with hexyl moieties was prepared using *n*-hexylamine (598 mg, 5.90 mmol) and TDT (1.05 g, 1.99 mmol) in a similar manner with OTSH (1.40 g, 1.69 mmol, 84.8%).

^1^H-NMR (CDCl_3_/CF_3_CO_2_H = 5:1, rt, *σ* in ppm): 0.90 (9H, t, *J* = 16 Hz, -C*H*_*3*_), 1.32 (18H, m, -(C*H*_*2*_)_3_CH_3_), 1.59 to 1.66 (9H, -SH and -C*H*_*2*_(CH_2_)_3_-), 2.94 (6H, br, -C*H*_*2*_SH), 3.30 to 3.41 (6H, br, -NHC*H*_*2*_CH_2_-), 4.11 to 4.47 (6H, br, -C*H*_*2*_CH(CH_2_SH)O-), 5.75 (3H, br, -CH_2_C*H*(CH_2_SH)O-), 8.06 (3H, br, -N*H*-). ^13^C-NMR (CDCl_3_/CF_3_CO_2_H = 5:1, rt, *σ* in ppm): 13.65 (−*C*H_3_), 22.42 (−*C*H_2_CH_3_), 25.91 (−*C*H_2_SH), 26.41 (−*C*H_2_CH_2_CH_2_CH_3_), 28.38 (−*C*H_2_CH_2_CH_3_), 31.28 (−NHCH_2_*C*H_2_-), 44.04 (−NH*C*H_2_-), 45.31 (−*C*H_2_CH(CH_2_SH)O-), 79.05 (−CH_2_*C*H(CH_2_SH)O-), 149.41 (*C*=O), 187.41 (*C*=S). IR (KBr, cm^−1^): 3,334 (NH), 2,573 (SH), 1,696 (C=O), 1,167 (C=S).

*IATSH*. TSH with isoamyl moieties was prepared using isoamylamine (526 mg, 6.03 mmol) and TDT (1.05 g, 1.99 mmol) in a similar manner with OTSH (644 mg, 817 μmol, 40.9%).

^1^H-NMR (CDCl_3_/CF_3_CO_2_H = 5:1, rt, *σ* in ppm): 0.91 to 0.95 (18H, d, *J* = 15 Hz, -CH(C*H*_*3*_)_2_), 1.43 to 1.48 (9H, -S*H* and -C*H*_*2*_CH(CH_3_)_2_), 1.60 to 1.63 (3H, m, -CH_2_C*H*(CH_3_)_2_), 2.91 (6H, br, -C*H*_*2*_SH), 3.19 to 3.43 (6H, br, -NHC*H*_*2*_CH_2_-), 4.17 to 4.47 (6H, br, -C*H*_*2*_CH(CH_2_SH)O-), 5.75 (3H, br, -CH_2_C*H*(CH_2_SH)O-), 8.03 (3H, br, -N*H*-). ^13^C-NMR (CDCl_3_/CF_3_CO_2_H = 5:1, rt, *σ* in ppm): 21.90 (−CH(*C*H_3_)_2_), 25.71 (−*C*H_2_SH), 26.70 (−*C*H(CH_3_)_2_), 37.06 (−*C*H_2_CH(CH_3_)_2_), 42.48 (−NH*C*H_2_CH_2_-), 45.42 (−*C*H_2_CH(CH_2_SH)O-), 79.10 (−CH_2_*C*H(CH_2_SH)O-), 149.50 (*C*=O), 187.50 (*C*=S). IR (KBr, cm^−1^): 3,323 (NH), 2,575 (SH) 1,696 (C=O), 1,176 (C=S).

*EHTSH*. TSH with 2-ethylhexyl moieties was prepared using -ethylhexylamine (773 mg, 5.99 mmol) and TDT (1.05 g, 1.99 mmol) in a similar manner with OTSH (1.43 g, 1.56 mmol, 78.2%).

^1^H-NMR (CDCl_3_/CF_3_CO_2_H = 5:1, rt, *σ* in ppm): 0.89 to 0.93 (18H, t, *J* = 18 Hz, -C*H*_*3*_), 1.30 to 1.38 (24H, m, -CH(C*H*_*2*_CH_3_) (C*H*_*2*_)_3_CH_3_), 1.57 (3H, br, -S*H*), 1.63 to 1.67 (3H, t, *J* = 19 Hz, -C*H*(CH_2_CH_3_) (CH_2_)_3_CH_3_), 2.94 (6H, br, -C*H*_*2*_SH), 3.17 to 3.54 (6H, br, -NHC*H*_*2*_-), 4.18 to 4.48 (6H, br, -C*H*_*2*_CH(CH_2_SH)O-), 5.77 (3H, br, -CH_2_C*H*(CH_2_SH)O-), 8.03 (3H, br, -N*H*-). ^13^C-NMR (CDCl_3_/CF_3_CO_2_H = 5:1, rt, *σ* in ppm): 10.37 (−CH(CH_2_*C*H_3_)(CH_2_)_3_CH_3_), 13.66 (−CH(CH_2_CH_3_) (CH_2_)_3_*C*H_3_), 22.86 (−CH(CH_2_CH_3_) (CH_2_CH_2_*C*H_2_CH_3_)), 23.98 (−CH(*C*H_2_CH_3_) (CH_2_CH_2_CH_2_CH_3_)), 26.08 (−*C*H_2_SH), 28.67 (−CH(CH_2_CH_3_) (CH_2_*C*H_2_CH_2_CH_3_)), 30.79 (−CH(CH_2_CH_3_) (*C*H_2_CH_2_CH_2_CH_3_)), 38.88 (−*C*H(CH_2_CH_3_) (CH_2_CH_2_CH_2_CH_3_)), 45.70 (−*C*H_2_CH(CH_2_SH)O–), 47.17 (−NH*C*H_2_-), 79.22 (−CH_2_*C*H(CH_2_SH)O-), 149.37 (*C*=O), 187.66 (*C*=S). IR (KBr, cm^−1^): 3,326 (NH), 2,573 (SH) 1,698 (C=O), 1,172 (C=S).

#### Polycondensation of TSHs and Zn(OAc)_2_ (typical procedure)

To a flask containing OTSH (268 mg, 201 μmol), a 1,4-dioxane solution (5.0 mL) of Zn(OAc)_2_ (55 mg, 300 μmol) was added under a nitrogen atmosphere. The mixture was stirred at 60°C for 24 h. The mixture was poured into an excess amount of methanol, and the precipitate was collected by filtration and drying under reduced pressure after washing with cold diethyl ether (131 mg, 91.7 μmol/unit, 45.3%).

^1^H-NMR (CDCl_3_/CF_3_CO_2_H = 5:1, *δ* in ppm): 0.88 (9H, t, *J* = 7.0 Hz, -C*H*_*3*_), 1.27 (90H, -(C*H*_*2*_)_15_CH_3_), 1.61 to 1.74 (6H, -CH_2_C*H*_*2*_(CH_2_)_15_-), 2.87 (6H, -CHC*H*_*2*_SH), 3.17 to 3.46 (6H, -NHC*H*_*2*_CH_2_-), 4.26 to 4.59 (6H, -NC*H*_*2*_CH-), 5.59 (3H, -CH_2_C*H*O-), 6.62 (3H, -(C=S)N*H*CH_2_-). ^13^C-NMR (CDCl_3_/CF_3_CO_2_H = 5:1, *δ* in ppm): 13.76 (−CH_2_*C*H_3_), 22.64 (−(CH_2_)_15_*C*H_2_CH_3_), 26.64 (−CH*C*H_2_S-), 29.18 to 31.98 (−CH_2_(*C*H_2_)_15_CH_2_-), 45.24 (−N*C*H_2_CH-), 49.75 (−NH*C*H_2_(CH_2_)_15_-), 76.54 to 77.17 (−CH_2_*C*HO-), 149.15 (*C*=O), 183.28 (*C*=S). IR (KBr, cm^−1^): 3,344 (NH), 1,697 (C=O), 1,160 (C=S).

Other TSHs were also polymerized in the same procedure:

(1) BTZnS: yield = 64%, IR (KBr, cm^−1^): 3,393 (NH), 1,696 (C=O), 1,160 (C=S).

(2) HTZnS: yield = 62%, IR (KBr, cm^−1^): 3,327 (NH), 1,696 (C=O), 1,163 (C=S).

(3) IAZnS: yield = 68%, IR (KBr, cm^−1^): 3,317 (NH), 1,698 (C=O), 1,171 (C=S).

(4) EHTZnS: yield = 62%, IR (KBr, cm^−1^): 3,374 (NH), 1,698 (C=O), 1,168 (C=S).

## Results and discussion

### Synthesis of TSH monomers

**Figure 1:**
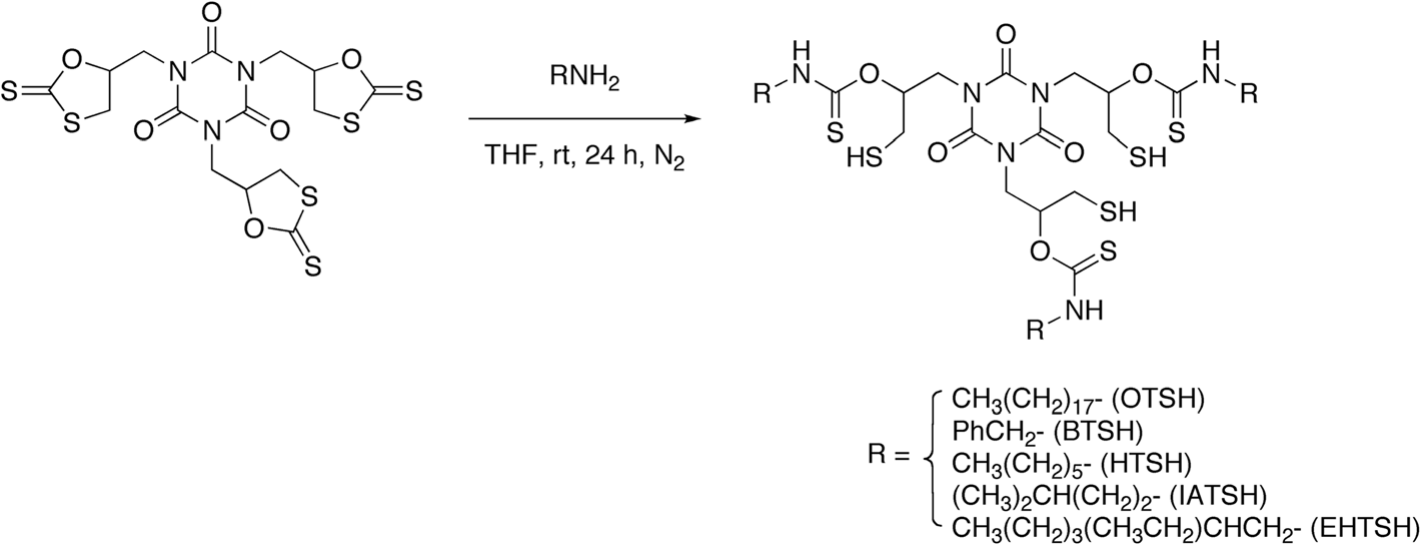
Synthesis of OTSH, BTSH, HTSH, IATSH, and EHTSH.

Five TSHs were prepared via the reaction of TDT with amines according to the previous report (Figure 1) [29]. The resulting thiols obtained from octadecylamine, benzylamine, *n*-hexylamine, isoamylamine, and 2-ethylhexylamine are abbreviated as OTSH, BTSH, HTSH, IATSH, and EHTSH, respectively. The isolated yields were moderate or good (OTSH 76%, BTSH 84%, HTSH 85%, IATSH 41%, and EHTSH 78%). OTSH, BTSH, HTSH, and IATSH are solid stably storable under air atmosphere, but EHTSH is an unstable viscous oil, which is gradually oxidized by oxygen.

### Polycondensation of TSHs and Zn(OAc)_2_

**Figure 2:**
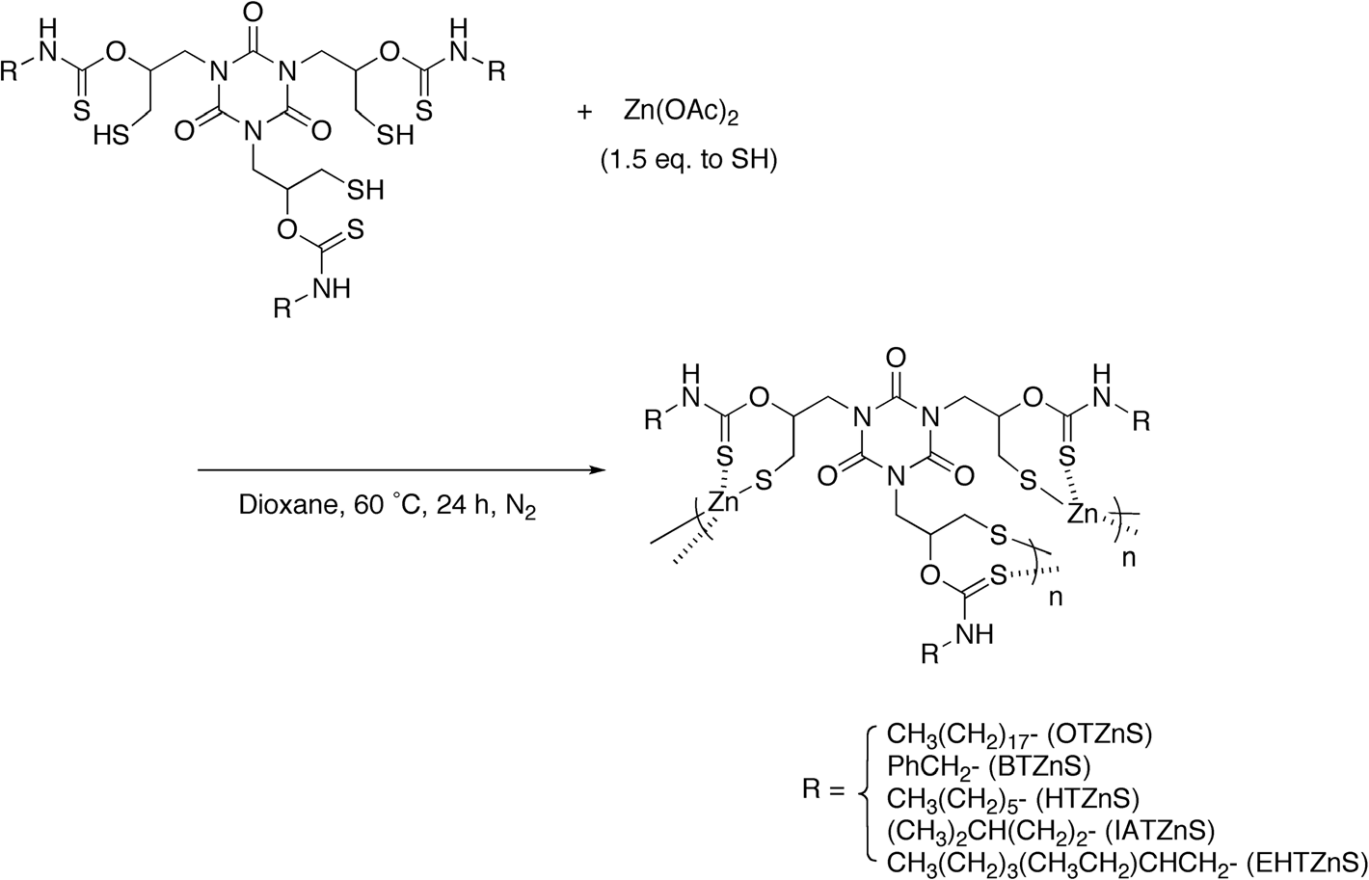
**Polycondensation of TSH and Zn(OAc)**_**2**_**.**

Polycondensation of TSHs with Zn(OAc)_2_ (1.5 equivalent to SH) was conducted in dioxane at 60°C for 24 h under a nitrogen atmosphere (Figure 2, Table 1). All the resulting reaction mixtures were homogeneous, and the white solids were obtained in 48% to 68% yields by precipitation into an excess amount of methanol. The resulting polymers are abbreviated as RTZnS in a similar manner with the abbreviation of the monomers. Only OTZnS having the long alkyl chains was soluble in common organic solvents such as THF and chloroform. HTZnS was slightly soluble in DMF, and the other products were insoluble in any common solvents. Plausible reasons for the poor solubility are cross-linking reactions, inherently poor solubility of the zinc complexes, and complexation with ZnO produced as a byproduct (discussed later).

**Table 1:** **Polycondensation of TSH and Zn(OAc)**_**2**_

**Run**	**Monomer**	**Yield (%)**^**a**^	***M***_**n **_**(*M***_**w**_**/*M***_**n**_**)**^**b**^	**Zn/S**^**c**^
1	OTSH	48	7400 (1.4)	0.29
2	BTSH	64	-^d^	0.40
3	HTSH,	62	-^d^	0.37
4	IATSH	68	-^d^	0.45
5	EHTSH	62	-^d^	0.71

Conditions: TSH 0.200 mmol, Zn(OAc)_2_ 0.300 mmol, dioxane 5.0 mL, 60°C, 24 h, N_2_. ^a^Isolated yield after precipitation with methanol. ^b^Estimated by GPC (THF, polystyrene standard). ^c^Estimated using EDX (ratios calculated as averages of ten spots). ^d^Not measurable due to poor solubility.

**Figure 3:**
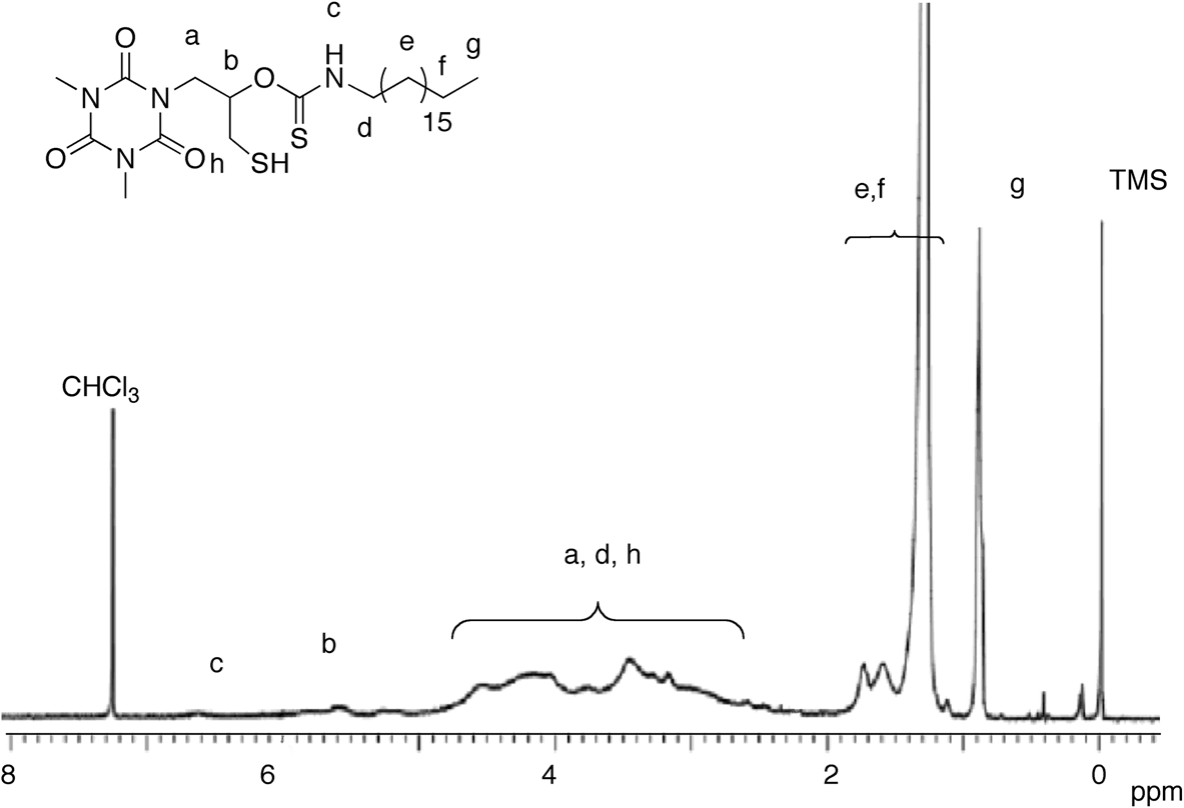
^**1**^**H-NMR spectrum of OTZnS (400 MHz, CDCl**_**3**_**/CF**_**3**_**COOH (*v*/*v *= 5:1)).** The assignment of the signals (a-h) is indicated on the structure.

**Figure 4:**
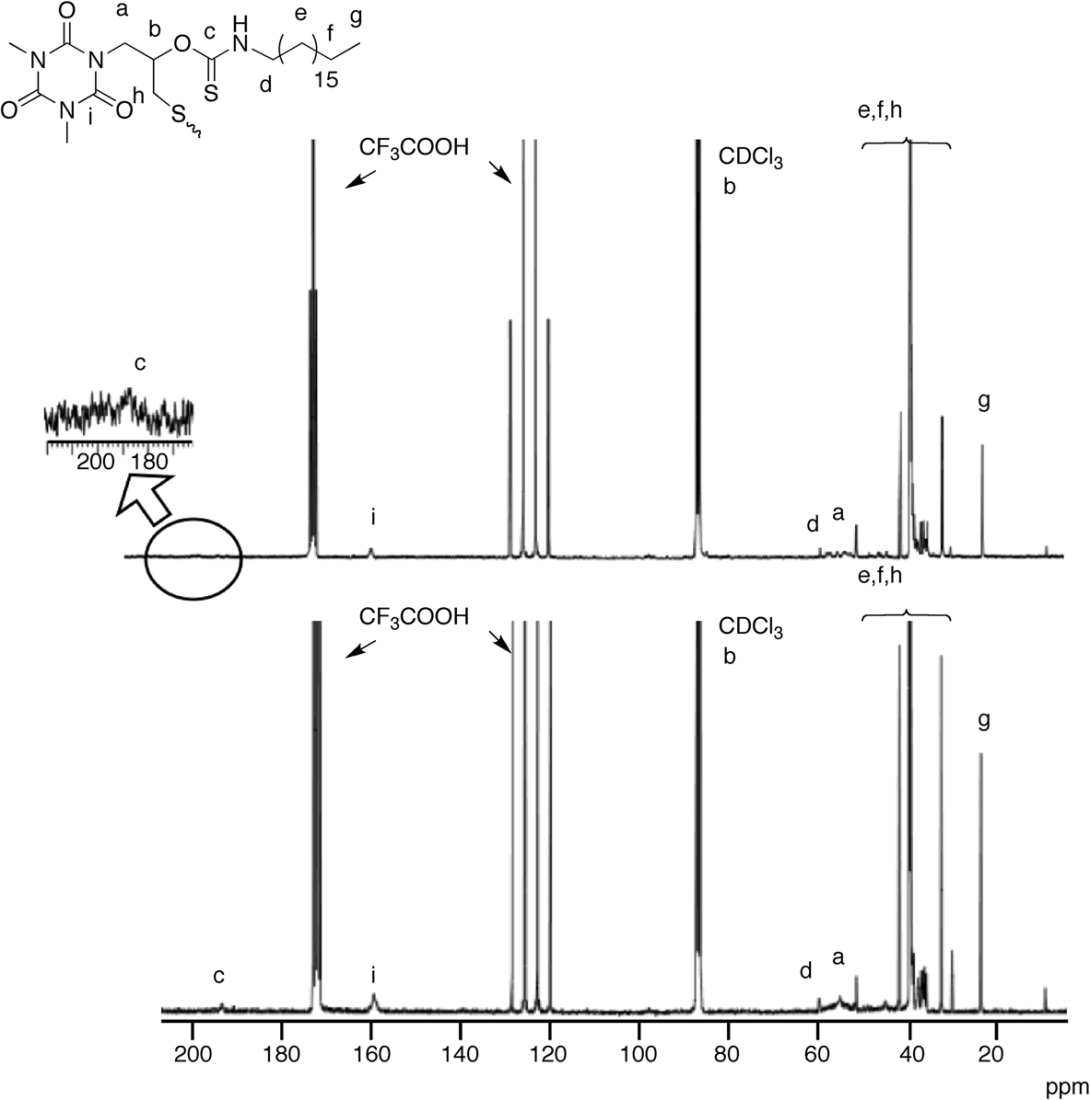
^**13**^**C-NMR spectrum of OTSH and OTZnS (100 MHz, CDCl**_**3**_**/CF**_**3**_**COOH (*v*/*v *= 5:1)).** The assignment of the signals (a-i) is indicated on the structures.

**Figure 5:**
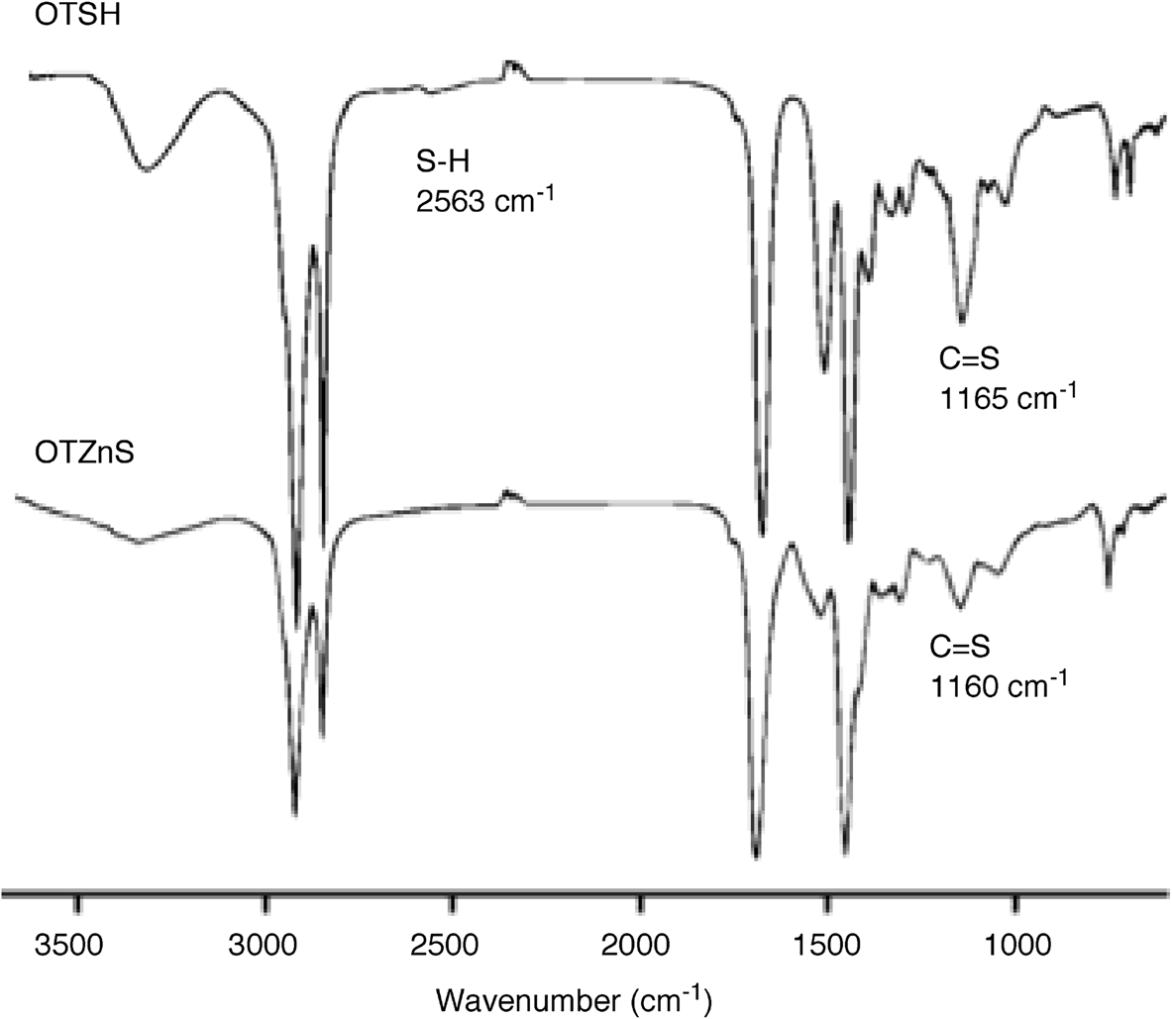
IR spectra of OTSH and OTZnS (KBr disks).

Structural characterization was conducted for OTZnS having enough solubility. The number average molecular weight (*M*_n_) was estimated to be 7,400, and the polydispersity index (*M*_w_/*M*_n_) was relatively narrow. The atom ratio of Zn/S estimated using EDX was 0.29 and almost agrees with the theoretical value (0.25). The quantitative elemental analysis by EDX was difficult for these powdery polymers, and the Zn/S values in this study may contain 20% to 30% of errors. The ^1^H-NMR spectrum showed signals at the regions agreeable to the expected structure, but was not informative enough for the elucidation of the structure due to the broad signals (Figure 3). The ^13^C-NMR and IR spectra were informative for its structural analysis (Figures 4 and 5). The IR absorption of the SH moieties at 2,564 cm^−1^ observed in the IR spectrum of OTSH was not observed in the IR spectrum of OTZnS, suggesting the formation of zinc thiolate structure. The ^13^C-NMR signals of -SCH_2_- carbons, -CH_2_NH-, and C=S carbons were shifted to lower magnetic field region by the transformation of OTSH into OTZnS, suggesting the changes in the structure around these moieties, whereas the other signals were observed at identical positions. The -SCH_2_- carbons in OTSH and OTZnS were observed at 26.4 and 29.4 ppm, respectively. The low-magnetic-field shift from the monomer to the polymer suggests the slight decrease in the electron density. Namely, this result suggests that -ZnSCH_2_- has a lower electron density than HSCH_2_-, although the small electronegativity of zinc implies that the zinc atom serves as a stronger electron-donating group than proton. Some ^1^H-NMR spectroscopic data were reported for zinc thiolates and their original thiols, and the chemical shifts were almost identical or the signals for zinc thiolates were observed at lower magnetic field regions [25,27]. A plausible reason is the backdonation from the occupied *d* orbital in zinc. The reason for the shifts of -CH_2_N- and C=S carbons can be ascribed to the coordination of the C=S group adjacent to the nitrogen atom onto the zinc atom. The coordination may also be confirmed by the IR spectrum. The absorption of the C=S moieties in OTZnS was observed at 1,160 cm^−1^, which were shifted from the absorption of OTSH at 1,165 cm^−1^. The low-wavenumber shift indicates the decrease in the *sp*^2^ character of the C=S moieties by coordination. Because other TZnS polymers were almost insoluble, their structures were elucidated by IR spectroscopy. The IR absorptions of the S-H bonds were not observable in all the IR spectra. Low-wavenumber shifts of the IR absorptions of the C=S bonds were observed in all the spectra. These data support the formation of the identical zinc thiolate structures.

The polycondensation of OTSH and Zn(OAc)_2_ was conducted under various conditions (Table 2). The effect of temperature was examined at 40°C to 80°C (runs 1 to 3). The yields were identical when the polymerization was conducted at 60°C and 80°C, but the yield decreased at 40°C, probably by the insufficient reactivity. The effect of concentration was not considerable. When the polycondensation was conducted in dioxane (12.5 to 37.5 L amounts toward 1 mol of OTSH), both the yields and the molecular weights were almost identical, but the higher concentration slightly increased the *M*_w_/*M*_n_ by the increase in the fraction with higher molecular weight (run 4). The increase of the high molecular weight fraction is attributable to the increased frequency of intermolecular coupling in this polycondensation of trifunctional and difunctional monomers. The polycondensation at 60°C under appropriately dilute concentration was proved to be the suitable conditions among examined. Although we tried polycondensation in the presence of tertiary amines to accelerate the condensation, the yield was not increased and the structure of the product became complex, probably by the undesired oxidative coupling of the thiol moieties. The hydrodynamic radius of the polymers determined by DLS indicated the nano-sized structure.

**Table 2:** **Polycondensation of OTSH and Zn(OAc)**_**2 **_**under various conditions**

**Run**	**Temperature (°C)**	**Dioxane (L/mol**_**OTSH**_**)**	**Yield (%)**^**a**^	***M***_**n **_**(*M***_**w**_**/*M***_**n**_**)**^**b**^	***R***_**h **_**(nm)**^**c**^
1	40	25	31	5,800 (1.4)	28
2	60	25	46	7,400 (1.4)	82
3	80	25	43	7,700 (1.6)	85
4	60	12.5	46	8,300 (2.1)	83
5	60	37.5	42	7,000 (1.6)	61

Conditions: OTSH = 0.200 mmol, Zn(OAc)_2_ = 0.300 mmol, 24 h, N_2_. ^a^Isolated yield after precipitation into methanol. ^b^Estimated by GPC (THF, polystyrene standards). ^c^Hydrodynamic radius determined by DLS (THF, 25°C, 1.3 g/L).

We considered the reason for the poor solubility of the products obtained from other TSHs. The IR spectra of the soluble and insoluble products were identical as aforementioned, suggesting that the side reactions are ignorable. This polymerization is a 2 + 3-type polycondensation and potentially yields cross-linked insoluble polymers. Intermolecular coupling reactions should be adequately suppressed to obtain soluble products. We presume that longer alkyl groups are advantageous not only to increase the solubility but also to suppress intermolecular coupling reactions. As a result, OTSH, having the longest alkyl group among examined, could give soluble polymers, whereas other TSHs could not due to the shorter alkyl chains insufficient to overcome these factors. The Zn/S values of the insoluble products were higher than the theoretical values. The higher Zn content implies the self-condensation of Zn(OAc)_2_ to produce oligomeric ZnO [30], which is also responsible for the insolubility. All the reaction mixtures after the reactions were homogeneous, and we presume that the self-condensation may have occurred during the purification processes.

### AFM analysis

**Figure 6:**
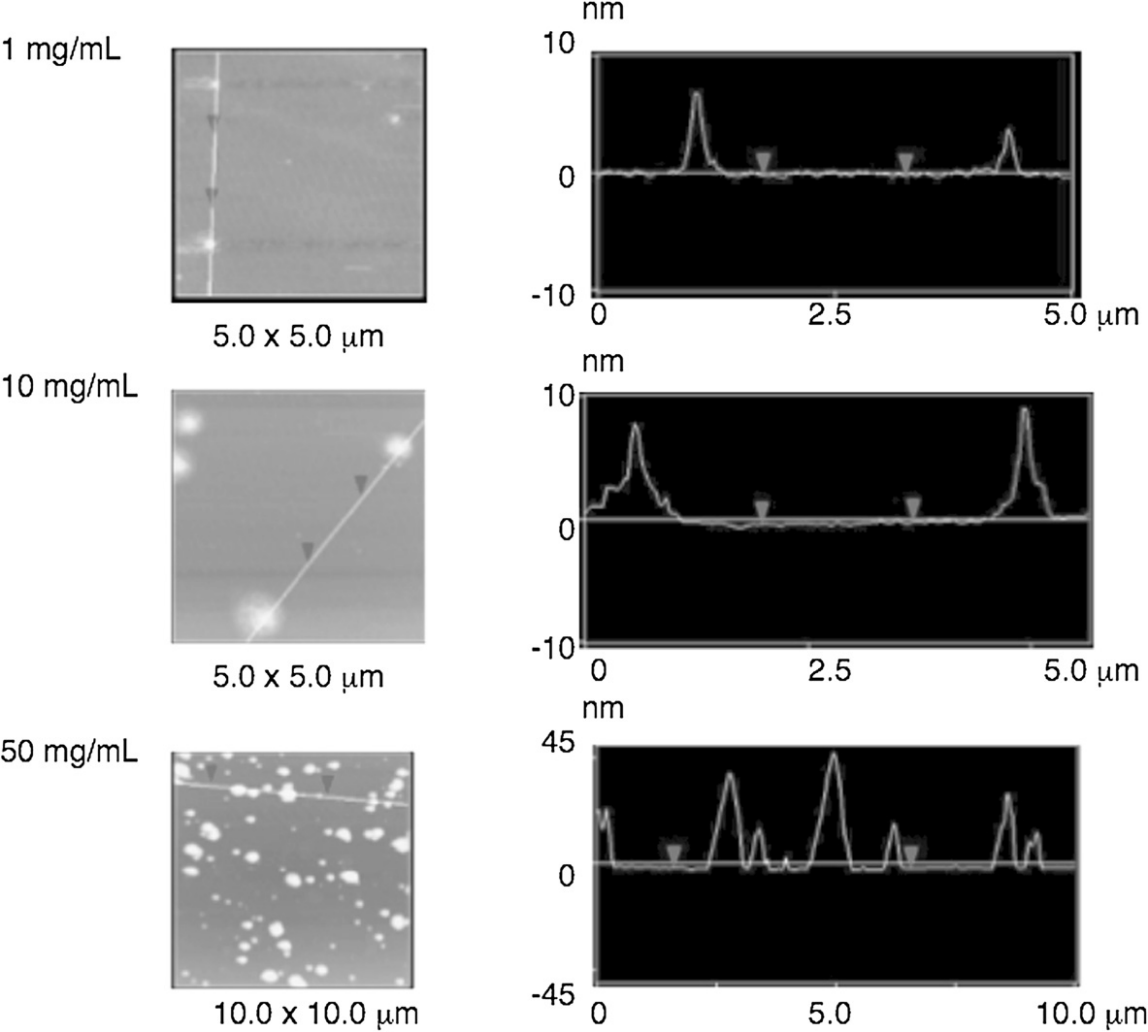
**AFM height and cross-sectional images of OTZnS obtained in run 1 in Table**2**.** Cast from 1, 10, and 50 mg/mL of THF solution on mica.

The solid-state structure of OTZnS obtained at run 1 in Table 2 was evaluated using AFM (Figure 6). The samples were prepared by casting 1, 10, and 50 mg/mL of THF solutions onto the mica substrates. The AFM images of OTZnS prepared from diluted 1 and 10 mg/mL solutions showed the presence of spherical nanoparticles with 10-nm height. Aggregated structures were not observable in the images, and the height distributions were very narrow. The heights can be correlated to the molecular size of OTZnS in the solid state. The good dispersion ability probably originated from the long alkyl chains existing on the surface to prevent aggregation [31]. The AFM image of OTZnS prepared from 50 mg/mL solution showed larger particles produced by aggregation, but particles larger than 50 nm were not observed. The good dispersibility is suitable for ingredients for optical materials without scattering by large aggregates.

### Refractive property of OTZnS

**Figure 7:**
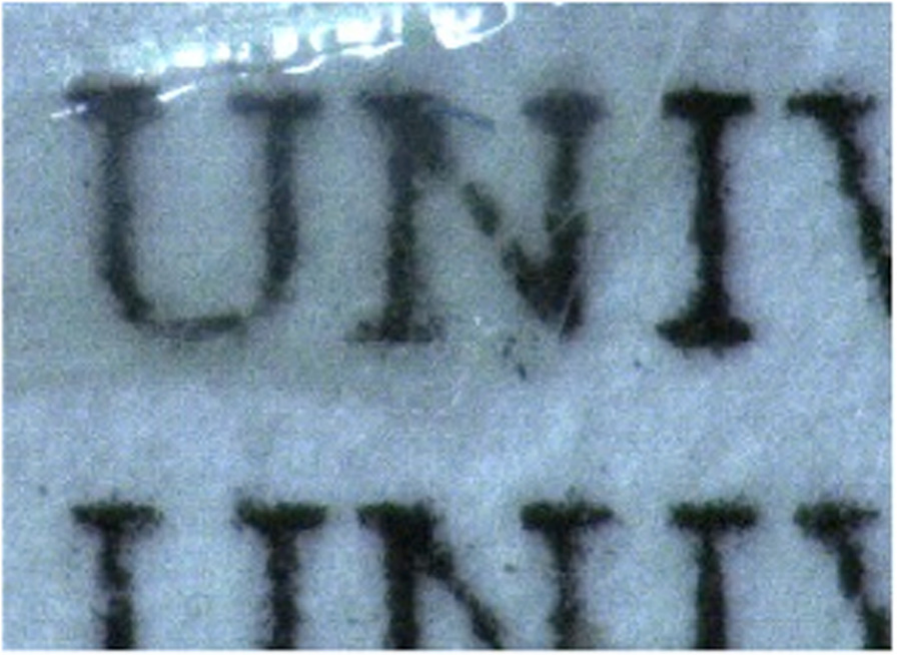
**Appearance of the composite film of OTZnS/PMMA (*w*/*w *= 67:33).**

The refractive property of OTZnS was evaluated. Unfortunately, the film cast from the solutions of OTZnS was very brittle and not self-standing enough for the measurement of refractive index. Accordingly, we evaluated the refractive indexes of the composite films of OTZnS and PMMA cast from the THF solutions (Table 3, Figure 7). The maximum weight composition of OTZnS was 67% for transparent film, and higher OTZnS composition resulted in the formation of brittle and heterogeneous films. The addition of OTZnS increased the refractive indexes of the resulting film, and the refractive indexes increased as the composition of OTZnS increased. The maximum *n*_D_ value reached 1.56, and the *n*_D_ value of OTZnS itself was calculated to be 1.58 by extrapolating the relationship between the *n*_D_ values and the compositions. This value is higher than that of OTSH (*n*_D_ = 1.53), indicating the efficiency of Zn to increase the refractive index. The *n*_D_ value of OTZnS is also higher than that of zinc acrylate having a higher Zn content (*n*_D_ = 1.42, Zn content of OTZnS = 6.9%, and Zn content of zinc acrylate = 31.5%). A plausible reason for the low *n*_D_ value of zinc acrylate is the low density originating from the long Zn-O bonds by the ionic character. Typical lengths of Zn-O bonds in zinc carboxylates are 2.0 Å [32-34] and those of the Zn-S bonds in zinc thiolates are 2.2 to 2.3 Å [24-27]. The bond lengths estimated from the single-bond covalent radius are 1.81 and 2.21 Å for the Zn-O and Zn-S bonds, respectively [35]. The significantly longer actual Zn-O bonds indicate the ionic character of the Zn-O bonds resulting in low densities, decreasing the refractive indexes. This result supports the validity of the design of this material, namely organic-sulfur-zinc hybrid materials, for refractive materials.

**Table 3:** Refractive indexes of OTZnS/PMMA film, PMMA film, and OTSH, and calculated refractive index of OTAnS

	**OTZnS/PMMA (*w */ *w *)**			**Calculated for OTZnS**	**OTSH**	**PMMA**
	**67:33**	**50:50**	**33:67**			
*n*_D_^a^	1.56	1.53	1.51	1.58	1.53	1.49

^a^Measured with Abbe refractometer at room temperature.

## Conclusion

A soluble organic-sulfur-zinc hybrid nanoparticle could be obtained by the polycondensation of OTSH and Zn(OAc)_2_. The resulting hybrid nanoparticle was miscible with PMMA and served as a refractive additive to increase the refractive indexes. The calculated *n*_D_ value for the polymer was 1.58. This value is relatively high as a compound bearing three octadecyl chains, and we believe that further optimization of the polymerization conditions will enable the synthesis of more refractive organic-sulfur-zinc materials with higher sulfur and/or zinc contents.

## Competing interests

Both authors declare that they have no competing interests.

## Authors’ contributions

BO and HK designed the study and were involved in writing the manuscript. HK carried out the experiments. Both authors read and approved the final manuscript.

## Authors’ information

BO received his Ph.D. degree in Polymer Chemistry in Tokyo Institute of Technology, Japan, in 2001. He is a professor in Yamagata University. His research activities include the development of organic-sulfur-inorganic hybrid materials, ion-conducting materials, and gene-delivery materials. HK was a Masters degree student at Yamagata University.
